# Online Delinquent Behaviors of Adolescents: Parents as Potential “Influencers”?

**DOI:** 10.1177/0306624X231206521

**Published:** 2023-10-30

**Authors:** Inge B. Wissink, Jessica J. Asscher, Geert-Jan Stams

**Affiliations:** 1 Utrecht University, The Netherlands; 2 University of Amsterdam, The Netherlands

**Keywords:** adolescents, online delinquency, cybercrimes, parenting, monitoring, internet addiction

## Abstract

In this study we examined whether aspects of parental monitoring of adolescents’ online behavior (rules regarding time spent on the internet, rules regarding content of internet use, frequency of communication, and quality of communication about internet use) are related to different kinds of online delinquent behaviors (sexting, spreading viruses, DDoS attacking, hacking, and online threatening) and whether the level of adolescents’ problematic (addictive) internet use mediates these relations. In regular Dutch high schools 1,009 adolescents filled out an online questionnaire (with adjusted versions of the ISPP, PIUQ, and the Dutch Youth Crime Monitor). Descriptive statistics showed that, in general, parents do not seem to monitor adolescents’ online behavior to a great extent. Furthermore, results of logistic regression analyses and mediation tests showed that fewer rules about online time, more rules about online content, and a good quality of parent-adolescent communication about online behavior are all associated with lower problematic internet use, which in turn is associated with lower odds of several online delinquent behaviors (mediation). Moreover, parental handling of rules about online content is also directly associated with lower odds of spreading viruses, hacking, and online threat.

Little is known about online delinquent behaviors of adolescents, compared to the attention for their offline (or traditional) delinquent behaviors, even though adolescents spend a substantial part of their time online (even more than they do at school) and statistics indicate that online delinquent behavior committed by juveniles is rising (Dutch National Police, 2019; van der Laan et al., 2021). In order to prevent future harm for society and juveniles involved, it is important to intervene timely and adequately. However, since cyber or online offences are a relatively new phenomenon, it is not completely clear how the development of online delinquency can be prevented and how different risk factors are related. The current study aims to further explore the role of parental factors (via monitoring of online behavior) in the explanation of several online delinquent behaviors and whether these relations are mediated by adolescents’ level of problematic (addictive) internet use. Our findings will help to unravel the possibilities for preventing or redirecting juvenile pathways to online delinquency.

As we all know, since the nineties, the use of the internet, smartphones, tablets, social media, and all kinds of web-based applications has risen dramatically. It has become a new part of everyday life, particularly for adolescents, as they are the ones who use these new forms of communication the most (Mishna et al., 2010).These developments have not only provided numerous favorable possibilities for adolescent development, but also some negative. One of them is that adolescents get more opportunities to behave in a delinquent and antisocial way and it is assumed that adolescents cross boundaries even more easily than they would do offline, mainly due to the anonymous nature of cyberspace. Notwithstanding these developments, only a few studies have been conducted on juvenile online delinquent behavior. Also, available studies mainly focused on factors that explain *victimization* of online delinquent behaviors rather than on factors that explain *perpetration* of online delinquency (Machimbarrena et al., 2018; Marcum et al., 2010; Mitchell et al., 2011; Montiel et al., 2016; van den Eijnden et al., 2010). Additionally, in many studies diffuse or generalized measures of “online delinquent behavior” have been used as a dependent variable, while differences between specific types of online delinquent behaviors are highly likely (Kim & Han, 2021). For instance, perpetrators of hacking (i.e., a cyber-dependent type of online delinquency) may show different risk factors than perpetrators of sexting or online threat (i.e., cyber-enabled types of online delinquency). Therefore, the current study will provide more information about possible differences between specific types of online delinquent behaviors (both in terms of prevalence and explaining factors). The study will also provide information about how parents try to monitor adolescents’ online behavior and, as already indicated, whether these parental online monitoring efforts (of adolescents’ online behavior) are related to the specific types of adolescents’ online delinquent behaviors. A final aim is to examine whether the expected relations between parental online monitoring efforts and adolescents’ online delinquent behaviors are (partly) mediated by adolescents’ problematic internet use.

In the “traditional” (offline) adolescent development literature the concept of “parental monitoring” was emphasized as an important guiding principle and protective factor in adolescent development (Kerr & Stattin, 2000; Stattin & Kerr, 2000). Monitoring was defined as parental awareness, watchfulness, and supervision of adolescent activities in multiple domains and communication to the adolescent that the parent is concerned about and aware of those activities (Dishion & McMahon, 1998). Not much is known about how parents try to monitor the online behaviors of their adolescents (further: parental online monitoring). It is assumed that parents are still learning in this respect as it is a relatively new aspect of parenting. Most of the current generation of parents are “newcomers” themselves to all the opportunities “the internet” provides, which means they also did not experience how their own parents approached this and whether that worked or not. In the current study four aspects of parental online monitoring are examined: parental handling of rules regarding both time spent and the content of internet use and both the frequency and quality of communication about internet use (following van den Eijnden et al., 2010). While the first two tap more into the “regulatory-supervisory” aspects of monitoring, the final two tap more into the “relational” aspects (Kerr et al., 2003). Previous studies in which these aspects were examined did not clarify which aspects parents show the most to monitor adolescents’ online behaviors (van den Eijnden et al., 2010). What previous studies did show is that these parental online monitoring aspects were related to compulsive internet, social media, and game use of adolescents (or: cyber-*victimization*; Koning et al., 2018; Tur-Porcar, 2017; van den Eijnden et al., 2010; Wang & Qi, 2017). However, these studies did not focus on online delinquent behavior committed by adolescents (or: cyber *perpetration*). Obviously, more information is needed on the role of parental online monitoring and its association with adolescent online delinquent behaviors, and whether these associations differ between different types of online delinquent behaviors.

Studies examining adolescent development already showed that parental monitoring of offline adolescent behavior is one of the strongest predictors of offline delinquent and other problem behaviors (Keijsers, 2016; Loeber & Stouthamer-Loeber, 1986; Racz & McMahon, 2011). There has been some debate about whether this parental knowledge was the result of parental solicitation (actively asking and following the child) or of spontaneous child disclosure, but the results indicated that knowing about the child’s whereabouts was indicative of a strong parent-child relationship, which, in turn, prevented children from behaving in a manner their parents would not approve of (via an internalization of parental norms). This higher reluctance of adolescents to cross boundaries because of a strong social bond with the parents is also in line with Hirschi’s social control theory. However, it is unclear whether the same holds for *online* behaviors of adolescents. Therefore, we aim to find out whether it is important for parents to actively monitor and control what their adolescents are doing online (i.e., setting and handling online rules) and/or whether good communication between parents and adolescents about their online behavior (indicative of a strong parent-child relationship) is protective for adolescent perpetration of different kinds of online delinquent behaviors.

In sum and more specifically, we examine adolescents’ perpetration of five particular forms of online delinquent behavior: sexting, spreading viruses, DDoS-attacking, hacking, and online threat and relate it to parental rules regarding the time spent on the internet, rules regarding the content of internet use, the frequency of communication about internet use and the quality of communication about internet use (or: parental online monitoring). Moreover, because previous studies showed relations between these “new” parental online monitoring variables and adolescents’ level of problematic internet use (van den Eijnden et al., 2010), we wish to find out whether adolescents’ problematic level of internet use (partly) mediates the relations between the parental online monitoring aspects and adolescents’ online delinquent behaviors. In other words, we aim to examine whether aspects of parental online monitoring (like handling rules concerning the time and content of internet use, and communication about it) are associated with a less problematic level of adolescents’ internet use, which in turn is related to lower chances of online adolescent delinquent behaviors. Hereby, the level of problematic internet use encompasses three aspects: obsessive thinking about the internet and mental withdrawal symptoms caused by the lack of internet use, neglect of basic needs, and everyday activities and difficulties in controlling internet use (Demetrovics et al., 2016). In the analyses, we will also control for several background variables (adolescents’ gender, age, ethnic background, educational level, parental marital status, and familial financial problems).The findings will be relevant because, as already explained, parental online monitoring is a relatively new aspect of parenting, and there seem to be no clear guidelines yet. As a consequence, most parents seem to act rather intuitively or do whatever they consider the best, or the easiest. Some parents may not monitor the online behavior of their adolescent to a great extent and, as a consequence, adolescents may feel that no one really monitors their behaviors online properly, and that they can often “get away” with online deviant or even delinquent behaviors (without penalty or punishment). This could reduce barriers to show these behaviors. In addition, cyberspace could be perceived more and more as an “unlawful” place where no rules are handled, which could pave the way for an aggravation of online delinquent behaviors shown by adolescents. Of course, such a development would be detrimental. In other words, parents need guidelines on how to deal with online behavior of adolescents in order to prevent them from entering a problematic and perhaps cybercriminal path, and also to protect others from victimization. To attain such a goal, it is first important to find out more about how current parents are actually trying to monitor their adolescents’ online behavior, and about if and how these behaviors (besides adolescents’ problematic internet use) are related to different types of online delinquent behaviors of the adolescents. Can parents be important “influencers” of their adolescents, and lead them the way towards positive behavior online?

The online delinquent behaviors we study are very different in nature (sexting, spreading viruses, DDoS attacking, hacking, and online threat). Behaviors like spreading viruses, DDoS attacking, and hacking are types that have only arisen since the discovery of the internet (i.e., cyber-dependent crimes), while the other studied online delinquent behaviors (online threat and sexting) are considered as online versions of already existing types of offline (or: traditional) delinquent behavior (i.e., cyber-enabled crimes). It is unclear whether the differences in the behaviors are also associated with differences in the relative importance of the explaining factors, as these different online delinquent behaviors have not yet been studied within a single study. Thus, it will be interesting to find out whether the included variables equally explain the different types of online delinquent behavior. If so, this would underline the importance of that factor for the explanation of adolescents’ online delinquent behavior in general, and also for the focus of prevention and intervention programs. If not, this would indicate the need for different kinds of approaches or responses.

## Method

### Participants

A total of 38 high schools were approached of which seven schools (18%) responded in a positive manner and participated. Whether the approached schools decided to cooperate or not was not associated with the number of pupils or location in the Netherlands and was mainly due to factors like whether the school/contact person had time to cooperate or was in a relatively “busy” time period already (for instance, because of a move or merge with another school). Of the participating classes in each school, almost all adolescents filled in the questionnaire (i.e., only a small proportion, 2.6%, did not consent to participate and 3.1% did not finish the questionnaire after consent). More specifically, at the seven (regular education) schools, 1,071 adolescents clicked on the link to the online questionnaire (starting with an information and consent letter), under supervision of a teacher and/or research assistant. After clicking on the link, 28 students did not provide consent and 34 students did not complete the questionnaire. This resulted in a final sample of 1,009 adolescents with a mean age of *M*_age_ = 14.53 (*SD* = 1.19; range 12.1–18.2). The sample consisted of 50.5% boys and 49.5 girls. Adolescents with a migration background (13.7%) were somewhat underrepresented in the sample (compared to the percentage of adolescents with a migrant background in the Netherlands which is 25.4% in the 10–15 years age group). Additionally, 33.4% of the sample followed lower vocational training (“vmbo”), 17.6% higher educational training (“havo”), and 49.0% pre-university training (“vwo/gymnasium”; i.e., the higher educational level is somewhat overrepresented; CBS Statline, 2020–2021). The parents of 19.1% of the adolescents in the sample were divorced and 3% of the adolescents reported that the family had financial problems (i.e., that bills could not be paid).

### Procedure

This study was part of a larger research project examining online behavior of school-going adolescents and associated factors. The project complied with the guidelines formulated by the Ethics Review Board of the Faculty of Social and Behavioral Sciences, University of Amsterdam, the Netherlands and was approved (CDE-2017-7732). After ethics approval, schools were either approached through mail, phone, or face-to-face via a known contact person of one of the researchers. Next, each school contact person that was interested received a letter of information regarding the purpose of the study and the criteria to participate.

After consultation of the school and parent boards, seven schools agreed to participate. All parents of the adolescents in participating classes were informed about the research by the schools, via the communication channels that were commonly used for sending important messages to parents. Most schools used a personally addressed mail with a link to their online communication channel to inform parents. If parents wished to withhold their adolescent from participation in the research project they could fill in a short form and hand this over to an assigned teacher at school or they could send a short email to the school or researcher. Less than 2% of the parents refused participation.

At the day of data collection, the adolescents sat in their classrooms and received a link to the online questionnaire. The questionnaire started with an information letter indicating the aims and purpose of the study, time needed for participation, and explaining that participation was voluntarily and completely anonymous, followed by a question to confirm that the adolescents had read the information and, if so, if they were willing to participate. Most adolescents were willing to participate (see Participants section for more information). During the time the adolescents filled in the questionnaire, an informed teacher (with phone support of a research assistant) or a research assistant was present. To make sure that the data were collected under similar circumstances a protocol was developed in which the complete procedure was written down. This protocol was used by the teachers and research assistants that were present during data collection.

### Measurements

#### Independent Variables

##### Control Variables

Several control variables were included: gender, age, ethnic background, educational level, relationship status of parents, and financial problems. Gender was coded as: 0 = *Boys*; 1 = *Girls*. Age was measured as a continuous variable. For ethnic background, adolescents were considered to have a migrant background if they themselves, or at least one of the parents was born outside the Netherlands (0 = *Migrant ethnic background*; 1 = *Non-migrant ethnic background*). Educational level was also dummy-coded (0 = *More practical educational level/lower vocational training*; 1 = *More theoretical level/higher general secondary and pre-university education*). As an indication of the relationship status of the parents adolescents filled in whether their parents were *Together* (0) or *Divorced* (1) or *otherwise related to each other* (missing). To get an indication of the presence of any financial problems within the householding the question “At your home, are there sometimes worries about bills that cannot be paid in time?” was used (1 = *Never*; 2 = *Rarely*; 3 = *Regularly*; 4 = *Often*; 5 = *Very Often*; and 6 = *Always*), which was recoded into a dummy-variable (0 = *No financial problems*; 1 = *Sometimes/more often financial problems*).

##### Parental Online Monitoring

To measure parental monitoring of the online behavior of the adolescents, we slightly adjusted the Internet Specific Parenting Practices (ISPP; van den Eijnden et al., 2010) to make it more in line with the latest developments (i.e., for instance new types of social media use were mentioned). With the resulting measurement we measured four aspects of parental online monitoring (POM): Rules with regard to time spent on the internet (RT; five items; α = .78; example item: “My parents allow me to go online -internet/snapchat/gaming etcetera—as long as I want to.” ; reversely coded so that a higher score represented a higher level of parental online monitoring), Rules with regard to content of internet use (RC; three items; α = .78; example item: “My parents allow me to do whatever I like online.” ; reversely coded so that a higher score represented a higher level of parental online monitoring), Frequency of communication about internet use (FC: three items; α = 77; example item: “How often do you and your parents talk about who you have internet contact with?”), and Quality of communication about internet use (QC; three items; α = .85; example item: “When my parents and I talk about my online activities I feel understood.”). A 5-point answering scale was used for RT, RC, and QC (1 = *Totally not true*; 2 = *Not true*; 3 = *Sometimes*; 4 = *True*; and 5 = *Totally true*) and for FC (1 = *Never*; 2 = *Sometimes*; 3 = *Regularly*; 4 = *Often*; and 5 = *Very often*).

##### Problematic Internet Use (PIU)

A translated version of the Problematic Internet Use Questionnaire Short Form (PIUQ-SF-6; Demetrovics et al., 2016) was used (six items; α = .72). Example items are: “How often do you spend time online when you’d rather sleep?” and “How often does it happen to you that you wish to decrease the amount of time spent online but you do not succeed?” and a 5-point answering scale was used (1 = *Never*; 2 = *Rarely*; 3 = *Sometimes*; 4 = *Often*; and 5 = *Always/almost always*).

#### Dependent Variables

To measure the online delinquent behaviors (dependent variables) seven items of the Dutch Youth Crime Monitor (YCM; Beerthuizen et al., 2020) were used to measure online delinquent behaviors. The introducing text was: “The following questions are again about your behavior online or on social media. Please indicate whether you have never or sometimes (once or several times) done the things below?”. A 2-point answering scale was used (0 = *No, never*; 1 = *Yes, I have sometimes done that*) to make it easier to fill in for adolescents and prevent missing data. In Table 1, the precise items that were used to measure all dependent variables and the corresponding frequencies are shown. For hacking and online threat a composite variable was computed (i.e., with score 1 if they had a score 1 on any of the two hacking/online threat items).

**Table 1.** Overview of Dependent Variables.

Dependent variable	Item	%^a^
- Sexting	Spreading sexual pictures or videos of someone younger than 18 years.	4.0
- Hacking changing password	Changing someone’s password, so that he/she could no longer log on.	11.1
- Hacking s/deleting data	Logging on someone else’s computer or profile and changing or deleting data, without that person knowing it.	5.1
- Spreading viruses	Deliberately sending around viruses to other computers using internet or e-mail.	2.4
- DDoS-attacks	Trying to shut down a website or mailbox by sending enormous amounts of information to it.	3.5
- Online threat by text, email, or chat	Sending someone a text, email, or chat message with the intention to scare him/her.	4.6
- Online threat by other social media	Sending someone a message using other social media like WhatsApp, Facebook, Twitter, Instagram, or Snapchat, with the intention to scare him/her.	6.3

a In this column the percentage of adolescents who indicated that they had ever shown this behavior (score = 1) is shown.

### Plan of Analysis

Descriptive statistics will be presented for the four parental online monitoring aspects and the online delinquent behaviors. Logistic regression analyses were performed to find out which independent variables were related to the various forms of online delinquent behaviors. In these analyses, at the first step the background variables were added (gender, age, ethnic background, educational level, parents divorced, and financial problems), at the second step the online monitoring subscales (RT, RC, FC, and QC), and at the final step the level of problematic internet use of the adolescent was added to the logistic regression model. Finally, we examined whether problematic internet use (PIU) fully or partly mediated the relations between RT, RC, FC, and QC and the five online delinquent behaviors using the PROCESS macro. In case of dichotomous dependent variables, PROCESS uses logistic regression for model estimation (instead of OLS regression; Hayes, 2022). With the PROCESS macro, for each independent variable X (RT, RC, FC, or QC), it was first tested whether that specific independent variable X was related to the mediator M (PIU). In these analyses, besides the control variables, the other online monitoring subscales were included as covariates. Next, PROCESS tests whether both the independent variable X and mediator variable M are related to the dependent variable Y (each online delinquent behavior separately). The PROCESS macro computes the total effect, along with the direct effect and the indirect effect of X on Y (on a log-odds metric) and the confidence intervals. If the confidence interval for the indirect effect is entirely above (or below) zero, this is evidence that the indirect effect is positive (or negative) to a “statistically significant” degree and it supports the conclusion that the indirect effect is positive (Hayes, 2022). The default number of 5,000 bootstrap samples was used (with 95% confidence intervals).

## Results

### Descriptive Statistics

First, to get more insight into how the adolescents perceived the four aspects of parental online monitoring and their levels of problematic internet use, the percentages are shown in Table 2. For the percentages of the different types of online delinquent behaviors, please see Table 1.

**Table 2.** Percentages of Online Parental Monitoring and Problematic Internet Use Statements.

Online parental monitoring (statements)	% True^a^
My parents allow me to go online as often as I want.	84.1
My parents allow me to go online as long as I want.	68.0
My parents have set a limit for the hours online.	21.5
My parents allow me to do online whatever I like.	65.8
My parents allow me to visit every online place I want.	67.0
My parents allow me to have online contact with anyone.	48.1
When my parents and I talk about my online activities I feel at ease.	89.3
When my parents and I talk about my online activities I feel understood.	79.6
When my parents and I talk about my online activities I feel taken seriously.	86.8
Online parental monitoring (questions)	% Never
How often do your parents say that you are not allowed to stay online much longer?	20.3
How often do your parents say that you are only allowed until a certain time?	43.4
How often do your parents say that you have to turn off the device?	13.5
How often do your parents talk about what you do online?	28.3
How often do your parents talk about the time spent online?	48.2
How often do your parents talk about your online contacts?	43.6
Problematic internet use (questions)	% Often-always
How often do you spend time online when you’d rather sleep?	46.2
How often do you feel tense, irritated, or stressed if you cannot use the internet for as long as you want to?	9.0
How often does it happen to you that you wish to decrease the amount of time spent online but you do not succeed?	17.3
How often do you try to conceal the amount of time spent online?	6.6
How often do people in your life complain about spending too much time online?	9.8
How often does it happen to you that you feel depressed, moody, or nervous when you are not on the internet and these feelings stop once you are back online?	4.2

a True = Cumulative percentage of categories: Sometimes—True—Totally true.

### Logistic Regression Analyses

#### Sexting

The results of the logistic regression analysis with sexting as a dependent variable showed that only the first model with the step with the background variables significantly added to the explanation of the odds of sexting perpetration (*p* < .001). Only gender and age were significantly related to the risk of sexting perpetration. The odds ratio of gender indicates that we observe a 68% decrease in the odds of sexting for girls. For each additional year of age, the odds of sexting increases by 69.5% (see Table 3).

**Table 3.** Logistic Regression Results for Sexting and Spreading Viruses.

Independent	Sexting		Spreading viruses	
	Model 1		Model 2	
	*B* (*SE*)	Exp (*B*)	*B* (*SE*)	Exp (*B*)
1				
Constant	−10.645*** (2.440)	0.000	−5.370 (4.389)	0.005
Gender	−1.140** (0.414)	0.320	−0.681 (0.610)	0.506
Age	0.528** (0.169)	1.695	0.211 (0.279)	1.235
Ethnic background	−0.520 (0.457)	0.595	1.136 (1.059)	3.114
Educational level	0.506 (0.551)	1.658	−1.218 (0.640)	0.296
Parents divorced	0.235 (0.428)	1.265	−0.057 (0.604)	0.945
Familial financial problems	0.681 (0.831)	1.975	0.529 (1.109)	1.696
2				
Rules time			0.329 (0.325)	1.390
Rules content			−0.831** (0.293)	0.436
Frequency communication			0.363 (0.269)	1.437
Quality communication			−0.269 (0.254)	0.764
3				
Problematic internet use				

*Note*. Model 1 Sexting *R*^2^ = .036 (Cox & Snell), .123 (Nagelkerke). Model X^2^(6) = 30.992***. Model 3 Spreading viruses *R*^2^ = .031 (Cox & Snell), .167 (Nagelkerke). Total model X^2^(11) = 26.731**.

*** *p* < .001. **p < .01.

#### Spreading Viruses

Only the second step significantly contributed to the explanation of spreading viruses (resp. *p* = .098, *p* = .015, *p* = .053; the significance of the first and third step was resp. *p* = .098, *p* = .053). In the second model, only parental rules about online content significantly explained the odds of spreading viruses (the more rules, the lower the odds; see Table 3 for the statistics of Model 2). The odds ratio’s in the final model indicate that if the RC score increases with one, we observe a 56.4% decrease in the odds of spreading viruses.

#### DDoS Attacking

For performing DDoS attacks the first (*p* = .001) and third steps (*p* = .002) were significantly contributing to the explanation. In the first model, only gender was significant (boys had higher odds). In the final model gender (higher odds for boys) and the level of problematic internet use (higher odds for adolescents with more problematic internet usage) contributed significantly to the explanation (see Table 4). The odds ratio’s of the final model indicate that we observe a 82.6% decrease in the odds of DDoS attack perpetration for girls. Finally, if the PIU score increases with one, the odds of DDoS attack perpetration increase with 140.6%.

**Table 4.** Logistic Regression Results for DDoS Attacking and Hacking.

Independent	DDoS attacking				Hacking			
	Model 2		Model 3		Model 2		Model 3	
	*B (SE)*	Exp(*B*)	*B (SE)*	Exp(*B*)	*B (SE)*	Exp(*B*)	*B (SE)*	Exp(*B*)
1								
Constant	0.969 (3.165)	2.636	−0.201 (3.259)	0.818	3.526* (1.791)	33.972	3.089 (1.807)	21.964
Gender	−1.648** (0.564)	0.193	−1.749** (0.566)	0.174	−0.435 (0.233)	0.647	−0.456 (0.233)	0.634
Age	−0.178 (0.198)	0.837	−0.276 (0.206)	0.759	−0.228* (0.114)	0.796	−0.266* (0.116)	0.767
Ethnic background	−.818 (0.465)	0.442	−0.787 (0.469)	0.455	0.275 (0.335)	1.317	0.331 (0.337)	1.392
Educational level	.213 (0.465)	1.238	0.388 (0.481)	1.474	−0.365 (0.247)	0.694	−0.333 (0.249)	0.717
Parents divorced	−.582 (0.570)	0.559	−0.614 (0.575)	0.541	−0.004 (0.269)	0.996	−0.014 (0.271)	0.986
Familial financial problems	−17.655 (7,893.325)	0.000	−18.033 (7,593.188)	0.000	0.220 (0.594)	1.247	0.078 (0.605)	1.081
2								
Rules time	−0.033 (0.266)	0.967	−0.197 (0.278)	0.821	0.024 (0.148)	1.024	−0.023 (0.151)	0.977
Rules content	−0.401 (0.217)	0.670	−0.356 (0.225)	0.701	−0.420*** (0.117)	0.657	−0.389** (0.119)	0.678
Frequency communication	0.314 (0.223)	1.369	0.308 (0.227)	1.360	0.010 (0.144)	1.010	−0.005 (0.145)	0.995
Quality communication	−0.007 (0.214)	0.993	0.214 (0.235)	1.239	−0.246* (0.110)	0.631	−0.184 (0.115)	0.832
3								
Problematic internet use			0.878** (0.275)	2.406			0.359* (0.151)	1.432

*Note*. Model 3 DDoS attacking *R*^2^ = .044 (Cox & Snell), .170 (Nagelkerke). Total model X^2^(11) = 37.880***. Model 3 Hacking.

*R*^2^ = .049 (Cox & Snell), .092 (Nagelkerke). Total model X^2^(11) = 42.533***.

*** p < .001.**p < .01. *p < .05.

#### Hacking

For hacking, all three steps were significant (resp. *p* = .005, *p* < .001, *p* = .019). In the first model, gender was significant (boys had higher odds). In the second model, age, parental rules about online content, and the quality of the communication were significant. In the third and final model, age, parental rules about online content and adolescents’ problematic internet use were significantly contributing to the explanation of the odds of perpetrating hacking (see Table 4 for the statistics of Model 2 and 3). The odds ratio’s in the final model indicate that, for each additional year of age we see a decrease in the odds of hacking of 23.3%. Also, if the RC score increases with one, we see a 32.2% decrease in the odds of hacking and, finally, if the PIU score increases with one, the odds increase by 43.2%.

#### Online Threat

For online threat the first significant step (*p* < .001) showed that gender, age, and educational level were significant. At the second significant step (*p* < .001), gender, age, educational level, and parental rules about online content were significantly contributing to the explanation of online threat. At the final third significant step (*p* < .001), gender, educational level, parental rules about content, and problematic internet use of the adolescent were significant with higher odds for perpetration of online threat for boys, more practically educated, adolescents with lower parental rules about online content, and with higher levels of problematic internet use (see Table 5 for the statistics of Model 2 and 3). The odds ratio’s in the final model indicate that we observe a 58.2% decrease in the odds of online threat perpetration for girls. Additionally, we see a 84.1% decrease in the odds of online threat for more theoretically (or: higher) educated adolescents. If the RC score increases with one, the odds of online threat decreases with 44.9% and, finally, if the PIU score increases with one, the odds increase with 119.5%.

**Table 5.** Logistic Regression Results for Online Threat.

Independent	Online threat			
	Model 2		Model 3	
	*B* (*SE*)	Exp(*B*)	*B* (*SE*)	Exp(*B*)
1				
Constant	−3.851 (2.377)	0.021	−5.456* (2.473)	0.004
Gender	−0.810** (0.306)	0.445	−0.872** (.312)	0.418
Age	0.327* (0.154)	1.387	0.283 (0.159)	1.328
Ethnic background	−0.544 (0.345)	0.580	−0.511 (0.352)	0.600
Educational level	−1.852*** (0.350)	0.157	−1.836*** (0.361)	0.159
Parents divorced	−0.119 (0.327)	0.888	−0.136 (0.336)	0.873
Familial financial problems	−0.855 (1.066)	0.425	−1.194 (1.075)	0.303
2				
Rules time	0.243 (0.185)	1.275	0.174 (0.192)	1.191
Rules content	−0.636*** (0.149)	0.529	−0.595*** (0.155)	0.551
Frequency communication	−0.134 (0.176)	0.875	−0.168 (0.179)	0.846
Quality communication	−0.020 (0.137)	0.980	0.151 (0.149)	1.163
3				
Problematic internet use			0.786*** (0.185)	2.195

*Note*. Model 3 Online threat *R*^2^ = .102 (Cox & Snell), .235 (Nagelkerke). Total model X^2^(11) = 91.522***.

*** *p* < .001. **p < .01. *p < .05.

#### Relation Online Parental Monitoring—Online Delinquent Behaviors: Mediation by Adolescents’ Problematic Levels of Internet Use?

**Figure 1.**
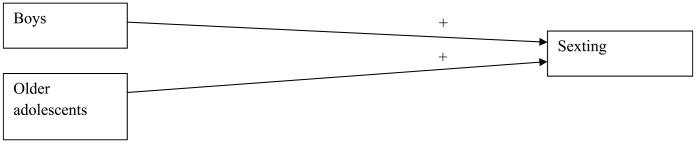
Sexting.

**Figure 2.**
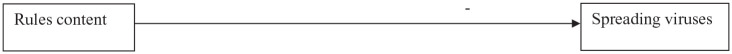
Spreading viruses.

**Figure 3.**
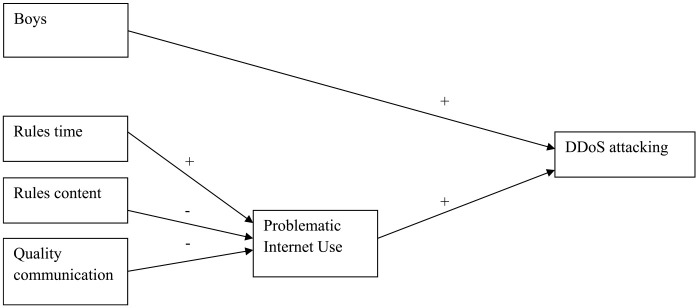
Online threat.

**Figure 4.**
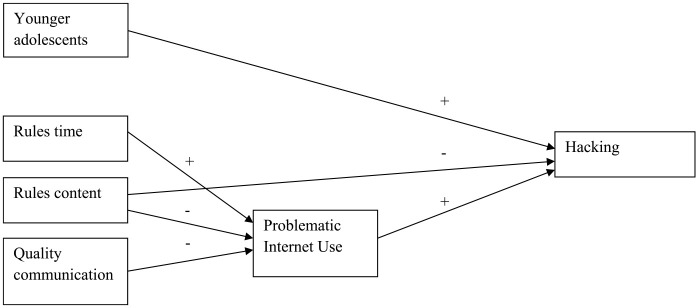
DDoS attacking.

**Figure 5.**
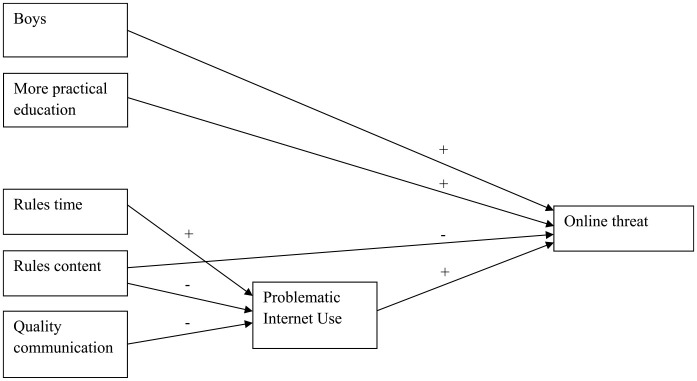
Hacking.

The mediation analyses were not performed for sexting and spreading viruses as for these types of online delinquent behavior, the final models with PIU were insignificant (see above; Figures 1 and 2). The results of the mediation analyses for the other online delinquent behaviors, first of all, showed that (while controlling for the background variables) both parental rules about online content (RC) and the quality of the parent-adolescent communication about the adolescent’s online behavior (QC) were significantly (*p* < .001) and negatively associated with PIU. So, the more parents handled rules about online content and had better communication with their adolescents, the lower the level of adolescents’ problematic internet use was. However, parents’ handling of rules about the time spent online was positively associated (*p* < .001) with PIU: the more parents handled rules about the amount of online time, the more adolescents showed problematic levels of internet use. Next, the mediation analyses indicated that for Hacking (H), PIU fully mediated the relation between both parental rules about online time (RT) and the quality of communication about online behavior (QC) on one hand and hacking (H) on the other hand (indirect relations) and partly mediated the relation between parental rules about online content (RC) and hacking (H) (as the direct relation was also significant, besides the significant indirect relation; Figure 3). The same was true for online threat (T; Figure 3). For DDoS attacking (D), PIU also fully mediated the relation between parental rules about online content (RC) and DDoS attacking (D), as it did for rules about online time (RT) and the quality of communication about online behavior (QC; Figures 4 and 5, Table 6).

**Table 6.** Overview of Results of Mediation Analyses (PROCESS).

Relation	Direct relations			Hypothesis	Indirect relations	
	Coefficient (*SE*)	*Z*	*p*		Percentile bootstrap 95% confidence interval	
					Coefficient	[Lower, upper]
Spreading viruses (V)						
RT->V	0.2613 (0.3327)	0.7855	.4322	RT->PIU->V	0.0780	[0.0078, 0.1848]
RC->V	−0.7961 (0.2972)	−2.6786	.0074	RC->PIU->V	−0.0684	[−0.1556, −0.0094]
FC->V	0.3137 (0.2732)	1.1484	.2508	FC->PIU->V	0.0078	[−0.0328, 0.0637]
QC->V	−0.1211 (0.2738)	−0.4423	.6583	QC->PIU->V	−0.1129	[−0.2367, −0.0124]
DDoS (D)						
RT->D	−0.1973 (0.2777)	−0.7105	.4774	RT->PIU->D	0.1150	[0.0457, 0.2180]
RC->D	−0.3356 (0.2247)	−1.5828	.1135	RC->PIU->D	−0.0949	[−0.1723, −0.0393]
FC->D	0.3077 (0.2268)	1.3567	.1749	FC->PIU->D	0.0086	[−0.0466, 0.0650]
QC->D	0.2140 (0.2347)	0.9117	.3620	QC->PIU->D	−0.1555	[−0.2639, −0.0775]
Hacking (H)						
RT->H	−0.0232 (0.1511)	−0.1533	.8782	RT->PIU->H	0.0472	[0.0100, 0.1001]
RC->H	−0.3892 (0.1186)	−3.2804	.0010	RC->PIU->H	−0.0382	[−0.0809, −0.0086]
FC->H	−0.0047 (0.1454)	−0.0322	.9743	FC->PIU->H	0.0036	[−0.0196, 0.0316]
QC->H	−0.1842 (0.1147)	−1.6062	.1082	QC->PIU->H	−0.0639	[−0.1241, −0.0159]
Online threat (T)						
RT->T	0.1744 (0.1918)	0.9093	.3632	RT->PIU->T	0.1026	[0.0465, 0.1866]
RC->T	−0.5954 (0.1546)	−3.8505	.0001	RC->PIU->T	−0.0840	[−0.1508, −0.0378]
FC->T	−0.1677 (0.1788)	−0.9381	.3482	FC->PIU->T	0.0072	[−0.0435, 0.0590]
QC->T	0.1513 (0.1493)	1.0139	.3106	QC->PIU->T	−0.1394	[−0.2244, −0.0768]

Thus, across most studied types of online delinquent behaviors (with the exception of sexting and DDoS attacks) a higher level of parental rules about the content adolescents could attend online was related to lower risks, also after adding adolescents’ level of problematic internet use, and while controlling for multiple background variables. This shows that adolescents’ problematic internet use does not mediate all relations between online parenting monitoring aspects and the online delinquent behaviors of adolescents. In other words, for the explanation of online delinquent behavior of adolescents, parental handling of rules about the content online has added value, besides it being associated with less problematic internet use of the adolescents. Parents with fewer rules about time spent online and a better quality of the parent-adolescent communication about the adolescent’s online behavior were related to a lower level of problematic (addictive) internet use which, in turn, was related to lower chances of spreading viruses, DDoS attacking, hacking, and online threat.

## Discussion

The current study was conducted to fill some gaps in the literature and to find out more about online delinquent adolescent behaviors, parental monitoring of adolescents’ online behavior (parents handling rules about online time and content and the frequency and quality of communication about adolescents’ online behavior) and problematic levels of internet use. Aims were to examine the role of parental monitoring of adolescents’ online behavior and the mediating role of problematic internet use in the explanation of the online delinquent behaviors, and to find out whether these associations are different when different online delinquent behaviors are examined.

Overall, adolescents with more problematic (addictive) levels of internet use had higher chances of having perpetrated three of the five examined cyberdelinquent behaviors (except sexting and spreading viruses): DDoS attacking, hacking, and online threat. Besides, one parental online monitoring aspect clearly stood out: parents’ handling of rules about what adolescents were allowed to do online (rules about online content). This factor was not only related to lower problematic levels of internet use, which in turn was related to lower chances of the online delinquent behaviors (indirectly), it was also directly related to lower chances of spreading viruses, hacking, and online threat (irrespective of the adolescents’ level of internet use). It is possible that, to prevent online delinquent behaviors, parental monitoring and restriction of what the adolescents are doing online is important. Surprisingly, the mediation analyses indicated that adolescents with parents handling more rules about online time more often showed higher levels of problematic internet levels. While it is possible that parents respond to problematic internet use by handling strict rules about time online, it is also possible that the handling of these kind of rules are counterproductive and have unwanted side effects. Perhaps adolescents with parents handling strict rules about time online will start to use it secretly, with higher risks of being online when they would rather go to sleep and for concealing their online time and other aspects that are indicative of problematic internet use (see Table 2). This may increase the risks for developing “hidden” and unmonitored spending of time online and, ultimately, for delinquent online behaviors. Longitudinal, qualitative and/or experimental research is certainly needed to further examine the relations between parents handling rules for online time and adolescent online delinquent behaviors. Either way, handling rules about online time was not directly related to online delinquent behavior (as parental handling of rules about online content was).

The results of the mediation analyses also showed that a better quality of the parent-adolescent communication about adolescent’s online behavior was associated with a lower problematic level of internet use, which in turn was associated with lower chances of having perpetrated spreading viruses, DDoS attacks, hacking, and online threat. It is possible that parents who are communicating well with their adolescents about their online behavior and who handle rules about online content stimulate adolescents to think about the temptations and dangers online and to use the internet for more positive social, constructive and stimulating activities and in more ethically or morally sound ways. Future research should confirm this suggestion. Weulen Kranenbarg et al. (2022) already indicated that it is important that schools of ICT-students provide lessons on the distinction between “good” and “bad” cyber-behavior and, in this way, encourage students to choose the positive alternative. Such a mindset may protect youngsters against the online “seductions” and from going astray online. However, it is also possible that another (third) variable explains these relations. Future studies could focus specifically on finding out more about these possible mechanisms behind the relations found for a good quality of communication about online behavior and especially for parental handling of rules about online content, because of the additional direct relations with online delinquent behaviors.

Another direction for future research is to include questions about parents’ online surveillance efforts (for instance behaviors like checking what the adolescent did online, watching along when the adolescent is online), as one of the components of monitoring. Also, questions about adolescents’ (spontaneous) disclosure about their online behavior could be included, or about the (offline) parent-adolescent relationship quality in general. To be better able to place the current results in line with the literature on offline monitoring, it is advised to include such questions in future research. Moreover, we should pay attention to the fact that our measures of the parental online monitoring aspects were asked in present terms, while the online delinquent behaviors were asked in terms of “Have you ever . . .” (i.e., lifetime online delinquency). As such, one could argue that it is possible that the delinquent behaviors have preceded parental online monitoring. However, our results do not seem to point in this direction as we consistently found a negative association between parental online monitoring (parental handling rules about online content) and online delinquent behaviors. It is less likely that parents have decreased their rules about online content in response to adolescents’ online delinquent behaviors than the other way around: that online delinquent behaviors decrease in response to higher parental online monitoring. Nevertheless, as already indicated, more longitudinal and/or experimental data are needed to find out more about the causal relations between parental online monitoring, adolescents’ internet behavior, and the risks in that behavior over time.

It is also interesting to find out whether the studied aspects of online parental monitoring are associated with other individual characteristics of the adolescents (besides adolescents’ disclosure; e.g., impulsivity). A previous study among school-going adolescents already indicated that adolescents with more depressive symptoms, who were anxious and with more general online risk behavior and online victimization were also at greater risk for online delinquent behaviors (Wissink et al., 2018). It is possible that there is a group of adolescents that is not too happy, who are spending a lot of their time online without parental monitoring, where they become victims (as a result of their risky behaviors), but where they may also become perpetrators. Our findings are in line with the suggestion, however, that parents can possibly be important “influencers” by preventing adolescents to develop into problematic internet users with hidden lives online (via rules about online content, fewer rules about online time and good communication about their online behavior) and, directly, by handling rules about what the adolescents are allowed to do online. The one exception seems sexting, as we found no significant relations between the studied online monitoring aspects and sexting perpetration. This is in line with other research findings suggesting that peer factors (like peer conformity) play a more important role in the explanation of sexting (Wissink et al., 2020). The report of Weulen Kranenbarg et al. (2022) offers more information about the importance of peer relationships (besides individual characteristics and environmental factors) for the explanation of cyber-delinquency among juveniles with ICT interests.

The current study showed that gender, age, and educational level turned out to be background factors that explained the examined online delinquent behaviors. Boys showed higher risks for perpetrating sexting, DDoS attacks, and online threats. Age differences were dependent on the specific online delinquent behavior: for sexting older adolescents had higher risks, while for hacking younger adolescents had higher risks. Finally, adolescents with more practical educational levels were more at risk for perpetrating online threats. Results of several studies suggest that adolescents from different educational levels have different risks depending on the specific online behavior and the level of technical knowledge that is needed for it (Spoormans, 2019; Wissink et al., 2020). Also, younger adolescents even seem more proficient in hacking than the older adolescents. Future research should focus on further examining the role of these background factors in the explanation of different online delinquent behaviors. The current study’s findings already demonstrate the importance of examining different kinds of online delinquent behaviors separately instead of looking at a single “aggregated” or global measure of “online delinquent behavior” or “cyber delinquency.”

Our findings regarding the important and unique role of both parental rules about online content and adolescents’ problematic levels of internet use raise concerns in light of our other findings about the frequencies of these behaviors. For instance, a high percentage of 66% of all adolescents indicated that it was true (varying from sometimes to totally) that their parents approved them to do online whatever they wanted. Additionally, almost half of all adolescents (46%) indicated that they often or (almost) always spend time online while they would rather sleep. As a previous study showed that adolescents themselves wished for more adult involvement in adolescents’ online behavior (in that case it concerned cyberbullying; Van der Meij, 2017; Wissink et al., 2018), we feel that it is important that parents remain involved with their adolescents and with what they are doing online. Our findings show that adolescents with parents who do not handle rules about what they do online or do not communicate with them about it, show more problematic internet use and, also, have higher risks for having perpetrated online delinquent behaviors. Future research could focus on confirming whether it is true that if parents extend or stretch their authoritative ways of parenting to the online domains, this protects adolescents against risks of online delinquent behavior perpetration. This is important, as adolescents will continue to explore the boundaries, and also, and perhaps even more so, in cyberspace.

### Limitations

The current study is not without its limitations. First, as already addressed above, both because the data were cross-sectional and because of the temporal ordering, experimental and/or longitudinal data are urgently needed to find out more about the causal paths. It is quite thinkable that a bi-directional model explains the relations between online parental monitoring and adolescents’ online behaviors (with parents reacting to the adolescent’s behavior and vice versa), just like it explains offline behaviors of adolescents and parents.

Second, the sample of the current study was not a true reflection of the Dutch adolescent population, because there were relatively few adolescents with a migrant background and lower or more practical educational level included (CBS Statline, 2020–2021). Unfortunately, Dutch practice turns out that especially schools with more adolescents with migrant backgrounds and lower educational levels are overloaded with requests for participation in scientific studies. As a result, these groups may sometimes be underrepresented in research.

Third, the parental online monitoring behaviors were reported by the adolescents. It is possible that parents do more to monitor the online behaviors of the adolescents than the adolescents are aware of. On the other hand, it is known that the way the adolescents perceive the behavior of their parents (such as rule setting and handling) is most important for the determination of their behavior (Kerr & Stattin, 2000). Reynolds et al. (2011) also showed that children’s and early adolescents’ reports of parental monitoring were more strongly predictive of later adolescent risk behavior than parents’ reports. Nonetheless, it would be interesting to also include observations and parental reports of their online monitoring behaviors in future research and compare them with the reports of the adolescents.

Additionally, the numbers of adolescents who reported to have committed the online delinquent behaviors were relatively low. With the interpretation of the percentages, however, one should keep in mind that it concerned a very young (mean age 14.5) and a relatively higher or more theoretically educated sample. Therefore, it is important to replicate the findings of the current study in a more diverse sample, with a higher representation of the lower or more practical educational levels, and a more ethnically mixed sample. Previous research findings also indicated that among adolescents in special educational schools and in residential settings online risk behaviors (such as sexting) are more prevalent as well (Middag et al., 2022; Wissink, 2021; Wissink & Kuipers, 2021). Future research should therefore also focus on these subgroups. Nevertheless, even the lower percentages of around 4% mean that in each class at least one adolescent (mean age 14.5; regular high schools; higher educated) has committed the concerning online offence which is forbidden by law and, therefore, punishable. Additionally, online delinquent behaviors that are already picked up at this young age (and that can develop without penalty) could be regarded as a first step in a later cybercriminal career. Recent statistics of the Dutch police and prosecution (December 2022) also confirm that cybercrime percentages are rapidly growing and that adolescents are increasingly involved in cybercrime. The police expressed their concern and stated that they see that it becomes easier to perpetrate cybercrimes, because ready-to-use tools are available to commit these crimes. They also mentioned the possibility that adolescents who commit cybercrimes may slide into regular criminal offences, which is worrisome. It is important to prevent such a development or intervene as early as possible. Although the lower numbers of adolescents who reported to have perpetrated the online delinquent behaviors may have impacted the validity of findings, the findings are very consistent across the different types of online delinquent behaviors. This strengthens confidence in the results. The variables that were found to be associated with the online delinquent behaviors showed a very consistent picture.

The present study has some important practical implications. Given that gender, age, educational level, parental handling of rules about internet content, and problematic internet use were all associated with online delinquent behaviors, this may provide important directions for prevention and intervention initiatives. It is recommended to target parents of especially boys, and preferably before they reach the age of late adolescence. Making these parents aware of the risks of problematic internet use and of the importance of handling rules regarding the content of internet use might be important steps. For interventions, our results indicate that parents are possibly important actors to address in order to prevent problematic internet use of their adolescents, teaching them to set rules about what the adolescents can do online and by communicating with their adolescents about their online behavior. In other words, it seems important to combine strictness and handling of rules with good communication (i.e., which is in line with authoritative parenting). Parents should also know that adolescents with less problematic levels of internet use and with parents who are handling rules about what adolescents are allowed to do online have lower risks for having perpetrated several kinds of online delinquent behaviors.
